# Digitization and Analysis of Capnography Using Image Processing Technique

**DOI:** 10.3389/fdgth.2021.723204

**Published:** 2021-10-29

**Authors:** Bhuwaneswaran Vijayam, Eko Supriyanto, M. B. Malarvili

**Affiliations:** ^1^School of Biomedical Engineering and Health Sciences, Universiti Teknologi Malaysia (UTM), Skudai, Malaysia; ^2^Institut Jantung Negara - Universiti Teknologi Malaysia (IJN-UTM) Cardiovascular Engineering Center, Universiti Teknologi Malaysia (UTM), Skudai, Malaysia

**Keywords:** capnography, capnometry, end tidal carbon dioxide, EtCO_2_, time based capnography

## Abstract

The study of carbon dioxide expiration is called capnometry. The graphical representation of capnometry is called capnography. There is a growing interest in the usage of capnography as the usage has expanded toward the study of metabolism, circulation, lung perfusion and diffusion, quality of spontaneous respiration, and patency of airways outside of its typical usage in the anesthetic and emergency medicine field. The parameters of the capnograph could be classified as carbon dioxide (CO_2_) concentration and time points and coordinates, slopes angle, volumetric studies, and functional transformation of wave data. Up to date, there is no gold standard device for the calculation of the capnographic parameters. Capnography digitization using the image processing technique could serve as an option. From the algorithm we developed, eight identical breath waves were tested by four investigators. The values of the parameters chosen showed no significant difference between investigators. Although there were no significant differences between any of the parameters tested, there were a few related parameters that were not calculable. Further testing after refinement of the algorithm could be done. As more capnographic parameters are being derived and rediscovered by clinicians and researchers alike for both lung and non-lung-related diseases, there is a dire need for data analysis and interpretation. Although the proposed algorithm still needs minor refinements and further large-scale testing, we proposed that the digitization of the capnograph *via* image processing technique could serve as an intellectual option as it is fast, convenient, easy to use, and efficient.

## Introduction

Capnometry is the expirometric study of the carbon dioxide gas. Although it is used interchangeably with “capnography,” “capnometry” means only the measurement of carbon dioxide (CO_2_) in respiratory gas without a continuous written record or waveform (1). The device in which both the capnometry and capnography are measured is called the capnometer. The capnometer is capable to sample both the time-based and volume-based signal (2). Time-based capnography is an expirogram that plots the expired CO_2_ partial pressure against a continuous duration of time (2, 3).

Capnography is a non-invasive tool that is known for its versatility. Traditionally it has been used by anesthetic and emergency clinicians to determine the placement of the endotracheal tube, monitoring depth of sedation, monitoring respiratory acidosis, and as a preventive measure to avoid CO_2_ narcosis. Over the years, the usage expanded toward the study of metabolism, circulation, lung perfusion and diffusion, quality of spontaneous respiration, and patency of airways outside of its typical utilization in the anesthetic and emergency medicine field (2).

Clinically, capnography usage may be utilized in both lung and non-lung diseases. For lung diseases, various research interest lies in bronchospastic spectrums such asthma, chronic obstructive pulmonary disease (COPD), cystic fibrosis and bronchopulmonary dysplasia (BPD) (4–7). There has also been the usage of capnography in pulmonary embolism (PE) (8, 9). For non-lung diseases, capnography has been a reliable tool to monitor several conditions. One prominent example would be the return of spontaneous circulation (ROSC) during cardiopulmonary resuscitation (CPR) as CO_2_ concentration monitoring is more reliable and sensitive than oxygen saturation (10). There are also other emerging usages of the capnography for congestive heart failure (CHF) (6). Another study has proven that it can be utilized as a primary low-cost yet efficient diagnostic tool to estimate the resting energy expenditure (REE) and resting metabolic rate (RMR) when compared with a calorimeter (3).

The capnometer which was once known as a critical care monitoring device has undergone evolution to its size and utilization of the side-stream technology, thus making it a robust diagnostic and screening tool (11). More parameters are now recognized and being utilized at both bench side and bed beyond the sole usage of End-tidal CO_2_ (EtCO_2_) in the capnometry. In 2016, Jaffe proposed that the capnographic analysis includes indices, slopes and angles, area, CO_2_ waveform measures and statistics, frequency transformations, Hjorth parameters, and Hjorth areas (12).

Currently, there are no established methods to digitize capnographic images for data analysis Jaffe (12), suggested that the computation of time and volumetric capnographic for analog signal could be done *via* the digitization of the paper tapes in which digital voltmeter and teletype paper punch signals would be traditionally imprinted. Therefore, here we propose capnography digitization and analysis using image processing technique as a means to bridge the analog to digital signal conversion for utilization by clinicians and researchers alike. Other than that, as previously mentioned by Tusman et al. (13), the normal values of most parameters and indices have not been established for non-invasive usage.

In this study, the digitization algorithm of the capnograph image would be scripted and optimized using computer software. These would be discussed in the methodology section.

## Materials and Methods

Generally, there were four main processes involved in the capnography image digitization and analysis. Initially, an image pre-processing was conducted. This was then followed by pixel indexing, signal analysis, and lastly displaying the results. These processes were carried out in MATLAB version 2018a (Mathworks, Natick, MA, US).

### Development of Algorithm

#### Pre-processing

The image pre-processing would be initiated by capturing, scanning, and transferring the time-based capnographic image in the Joint Photographic Expert Group (JPEG) or portable network graphic (PNG) file extension form to the desired folder of the user.

##### Skew Correction

The skew correction was a process in which the “y-” and “x-axises” were realigned to be parallel to the respective height and width of the image. This was previously suggested by a paper that digitized ECG images (14).

##### Determination of Y- and X-Axis

Secondly, the boundaries of the corresponding axis which exhibited the wave signal were determined. This resulted in two respective polygons that would be used to crop the y-axis, which was the concentration of the CO_2_ in mmHg, and the x-axis which was time in seconds. A user input dialog box was utilized to type in the values of the real distance of scale.

##### Re-scale and Resize the Image

The re-scaling and re-sizing were done according to the y- and x-axis which corresponded to the height of the image. The scaling process was done to accommodate changes of each pixel to represent each axis distance. The rescaling of the pixel distance was done *via* a factor between the pixel distance and the real distance of the scale.

##### Crop Breath Signal(s)

After re-sizing the image, the desired breath signals are now chosen *via* a “polygon” cropping function or tool. The polygon is then cropped out from the rest of the image for further processing. At this point, wave signals from neighboring breath waves that are unwanted may still be part of the exhibited image.

##### Filter Breath Signal Image

Afterward, the cropped breath signal was filtered to remove unwanted pixels. An example of this method is binarization which converts the foreground and background of the image to pixel values of 255 and 0, respectively (15). Other than that, additional filtering and image masking was carried out on noisy datasets and images with artifacts such as “salt and pepper” appearance after filtering to increase the signal-to-noise ratio (SNR) (16). A script was also utilized to automatically determine the start and of the expiration and the end of inspiration automatically to tidy up the cropping process from the section Crop Breath Signal (s).

##### Skeletonize Filtered Image

The final stage of the pre-processing was the skeletonization of the filtered signal breath wave. This resulted in a 1-pixel line foreground image with no redundant and repetitive data on the y- and x-axis (17).

#### Pixel Indexing

**Figure 1:**
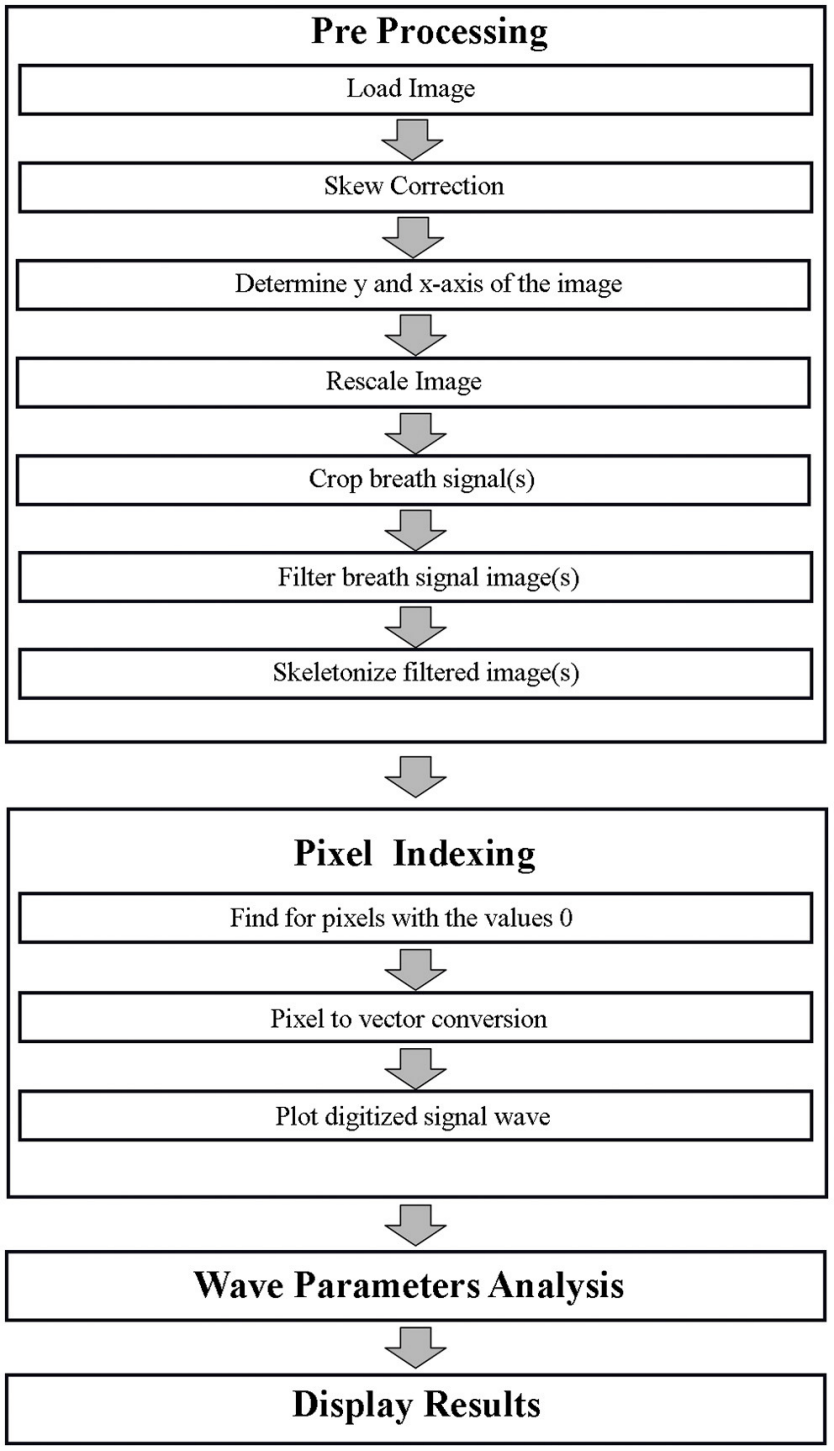
Capnographic image digitization algorithm.

The pixel indexing was the second stage of the capnography image digitization and analysis. This was conducted as recommended in the study by Naam in the digitization of ECG (17). Another similar study that was previously mentioned for the de-skewing process also utilized the same method in their algorithm (14). The skeletonized data are indexed according to pixels where black pixels are indexed as “0” whereas white pixels are “1.” The pixels with a value of “0” are then converted into vectors of the previously cropped image. This wave image to 1 dimensional (1D) signal conversion could be called the “digitization process” (16). The digitized vector could then be plotted to form an identical signal as the original capnographic image data. The algorithm flow for both pre-processing and pixel indexing is displayed as part of the flow chart in Figure 1.

#### Wave Parameters Analysis

The signal analysis was done with a few predetermined parameters. Although there are numerous capnometric parameters up to date, these specific parameters were chosen based on the clinical familiarity and past experiences of the authors. The parameters and their category from the proposed classification were explained as follow:

a) Carbon dioxide concentration and time points and coordinates
  i. EtCO_2_
  ii. Bohr's PACO_2_ (3, 13)
b) Slopes
  i. S1 (7)
  ii. S2 (7)
c) Angles
  i. Alpha Angle (18)
d) Volumetric studies
  i. Expiratory CO_2_ tidal volume per breath (VTCO_2_Br) (13, 19)
e) Functional transformation of wave data
  i. S2-S1 Slope Ratio (S2-S1SR) (7)
  ii. Expiratory to Inspiratory Ratio (ETIR)

Calculations were coded *via* functions and equations into MATLAB accordingly. The S1 was the gradient slope where the capnogram signal starts at 4 mmHg for 0.25 s on phase II of the capnogram (7, 20). Meanwhile, S2 was the gradient slope from 0.75 to 1.25 s (7, 21). The S2-S1 Slope Ratio (S2-S1SR) was calculated as follows (7, 12, 21):


$$\begin{array}{l} x=y/z\;\times 100;where\;x\;is\;S2-S1SR,\;y\;is\;S2\;and\;z\;is\;S1 \end{array}$$

The alpha angle was the angle between S1 and S2 (18, 22). The tidal elimination of CO_2_ per breath (VTCO_2_Br) was the integration of flow and CO_2_ signals over the entire breath cycle (19). One of the easiest estimations of Bohr's partial pressure of mixed alveolar CO_2_ (PACO_2_) was by determining the mid-point of the expiratory phase of the capnograph (23). The expiratory to Inspiratory Ratio (ETIR) was the duration ratio of the expiration and inspiration. The EtCO_2_ was the peak of the CO_2_ in mmHg.

### Algorithm Testing

Breath data of real patients from a previous study was plotted on MATLAB (24). A few CO_2_ concentration vs. time images were exported out as JPEG or PNG. Out of these images, eight breath waves were analyzed by four different investigators at our center by using the algorithm that we proposed earlier. The eight waves were determined *via* an agreement among the four investigators. The sharpness, appropriateness, signal condition, and clipping were considered before deciding on the final eight waveforms.

The results of all the capnometric parameters were tabulated and analyzed in MedCalc version 19.2.6 (MedCalc Software Ltd., Ostend, Belgium). All parameters were analyzed and tabulated as continuous numbers to three decimal points. To ease calculation, the ETIR was expressed in decimal points rather than a ratio.

An ANCOVA with the investigators as covariates was used to analyze the repeated capnometric parameters for differences. *P*-values lower than 0.05 were considered statistically significant.

## Results

### Capnographic Image Digitization

**Figure 2:**
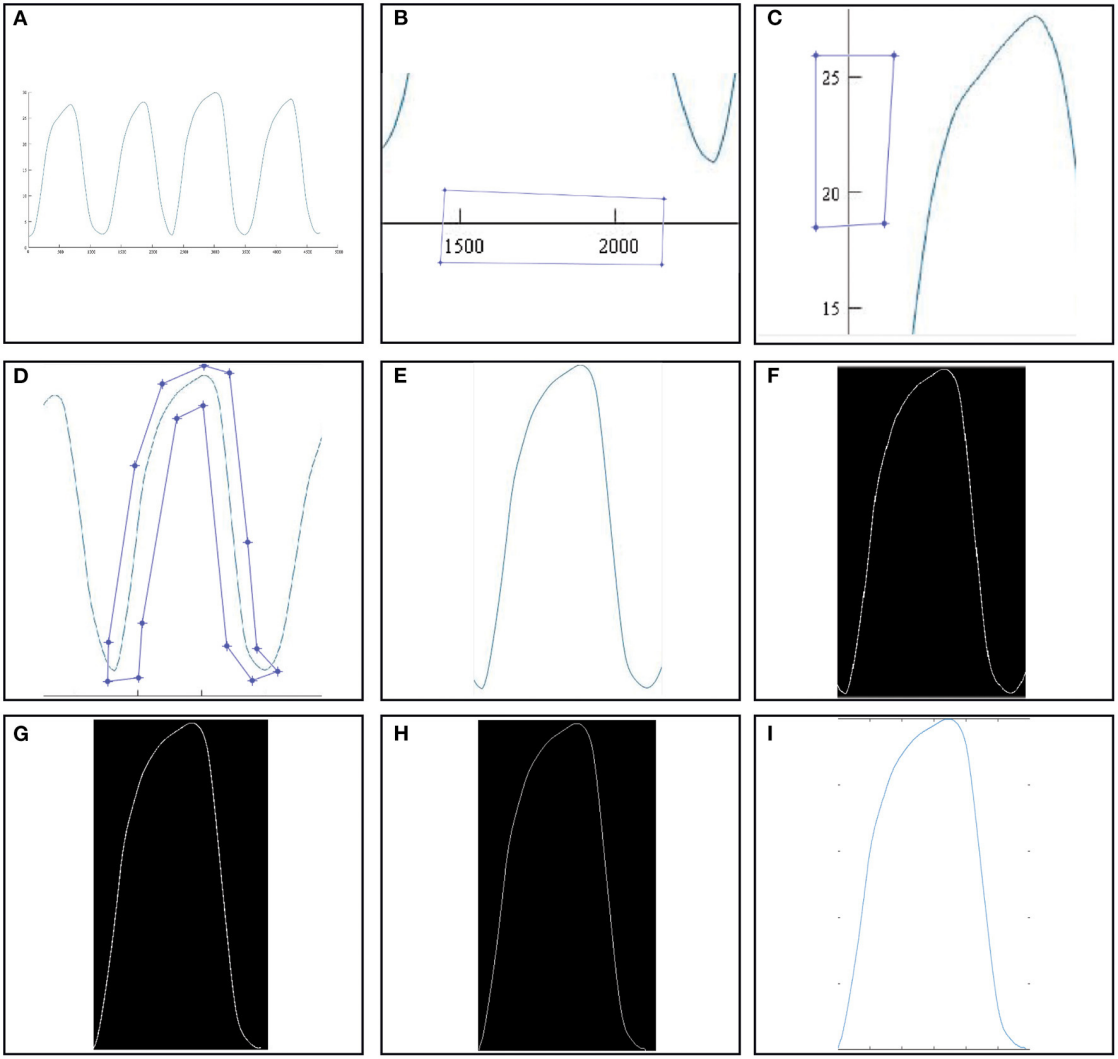
Image processing algorithm of an image with capnograph. **(A)** Raw capnographic signal. **(B)** x-axis cropping. **(C)** y-axis cropping. **(D)** Breath waveform cropping. **(E)** Cropped breath waveform. **(F)** Binarized crop yield from image **(E)**. **(G)** Duration optimized single breath waveform. **(H)** Skeletonized version of image **(G)**. **(I)** “Digitized” signal.

The algorithm as discussed in the method section of this study was tested and the results for each image processing technique utilized are shown according to its process. Figure 2 shows the fate of a single breath from an image that underwent processing in MATLAB and eventually making parametric extraction possible.

Image (A) in Figure 2 showed a raw image with a few capnograph waves. Images (B,C) showed the scaling process in which the x- and y-axes were sampled to determine the distance in pixels and the scaling factor. Furthermore, image (D) showed the desired breath signal to be sampled. The polygon method was chosen for cropping as shown in the image. Image (E) was the yield of the polygon crop method while (G) was the same image after binarization. Image (F) was the auto-optimized breath wave that begins with the start of expiration and ends with the inspiration. Image (H) was the skeletonized 1D version of image (G). Lastly, the image (I) was the digitized plotted signals of image (H). The “digitized” data could now be analyzed further according to CO_2_ concentration and time points and coordinates, slopes angle, volumetric studies, and functional transformation of wave data. The calculation of the parameters was done after obtaining such a signal on MATLAB.

**Figure 3:**
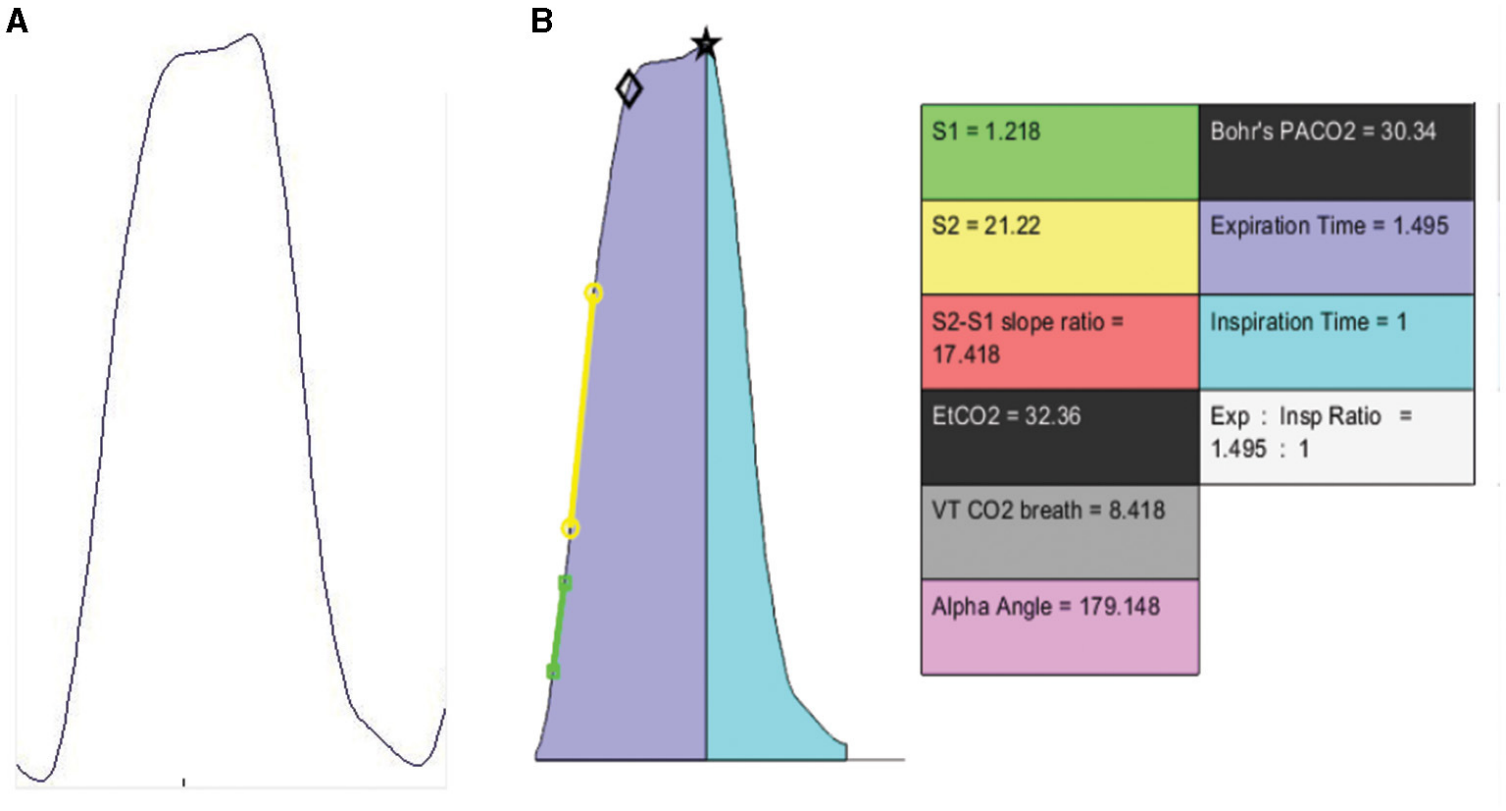
**(A)** Raw capnographic signal. **(B)** “Digitized” signal with derived capnometric parameters. The black star and diamond are MATLAB derived plot coordinates for EtCO2 and Bohr's PACO2 respectively.

An example of a raw and digitized breath signal displaying the capnometric parameters post-application of the proposed digitization algorithm is shown in Figure 3. The parameters that were mentioned in the earlier section are displayed as values on the right side of the image.

### Capnographic Signal Analysis and Algorithm Testing

A total of four investigators were able to apply the proposed algorithm on MATLAB. The parameters, *F*-ratio, and *p*-values are displayed in Tables 1, 2.

**Table 1:** The EtCO_2_, PACO_2_, S1, and S2 analysis.

	**EtCO_**2**_**	**PACO_**2**_**	**S1**	**S2**
Breath wave 01 [*F* ratio (*p*- value)]	3.000 (*p* = 0.225)	0.057 (*p* = 0.835)	3.000 (*p* = 0.333)	17.28 (*p* = 0.053)
Breath wave 02 [*F* ratio (*p*- value)]	3.000 (*p* = 0.225)	0.059 (*p* = 0.830)	8.333 (*p* = 0.212)	0.714 (*p* = 0.487)
Breath wave 03 [*F* ratio (*p*- value)]	8.000 (*p* = 0.106)	8.854 (*p* = 0.097)	0.143 (*p* = 0.742)	16.333 (*p* = 0.056)
Breath wave 04 [*F* ratio (*p*- value)]	8.000 (*p* = 0.106)	17.069 (*p* = 0.054)	^*^NC	7.348 (*p*=0.113)
Breath wave 05 [*F* ratio (*p*- value)]	8.000 (*p* = 0.106)	7.027 (*p* = 0.118)	5.069 (*p* = 5.069)	10.000 (*p* = 0.087)
Breath wave 06 [*F* ratio (*p*- value)]	2.946 (*p* = 0.228)	2.986 (*p* = 0.226)	2.999 (*p* =0.225)	2.996 (*p* = 0.226)
Breath wave 07 [*F* ratio (*p*- value)]	3.000 (*p* = 0.225)	2.999 (*p* = 0.226)	^*^NC	2.455 (*p* = 0.258)
Breath wave 08 [*F* ratio (*p*- value)]	3.000 (*p* = 0.225)	2.979 (*p* = 0.227)	2.995 (*p* = 0.226)	2.999 (*p* = 0.2255)

* *NC implicates non-calculable vales*.

**Table 2:** The S2S1Sr, VtCO_2_, alpha angle, and ETIR analysis.

	**S2-S1SR**	**VtCO_**2**_**	**Alpha angle**	**ETIR**
Breath wave 01 [*F* ratio (*p*- value)]	3.000 (*p* = 0.333)	10.229 (*p* = 0.085)	3.000 (*p* = 0.333)	3.000 (*p* = 0.225)
Breath wave 02 [*F* ratio (*p*- value)]	8.333 (*p* = 0.212)	12.017 (*p* = 0.074)	8.333 (*p* = 0.212)	3.820 (*p* = 0.190)
Breath wave 03 [*F* ratio (*p*- value)]	0.561 (*p* = 0.532)	3.177 (*p* = 0.217)	0.517 (*p* = 0.547)	8.000 (*p* = 0.106)
Breath wave 04 [*F* ratio (*p*- value)]	3.220 (*p* = 0.215)	3.220 (*p* = 0.215)	^*^NC	11.590 (*p* = 0.077)
Breath wave 05 [*F* ratio (*p*- value)]	6.698 (*p* = 0.123)	2.542 (*p* = 0.252)	9.567 (*p* = 0.091)	8.000 (*p* = 0.106)
Breath wave 06 [*F* ratio (*p*- value)]	2.973 (*p* = 0.227)	2.973 (*p* = 0.227)	2.948 (*p* = 0.228)	3.000 (*p* = 0.225)
Breath wave 07 [*F* ratio (*p*- value)]	6.067(*p* = 1.000)	2.993 (*p* = 0.226)	^*^NC	3.000 (*p* = 0.225)
Breath wave 08 [*F* ratio (*p*- value)]	2.908 (*p* = 0.230)	2.989 (*p* = 0.226)	2.966 (*p* = 0.227)	3.000 (*p* = 0.225)

* *NC implicates non-calculable vales*.

For the EtCO_2_, PACO_2_, S2, S2-S1SR, VtCO_2_, and ETIR, there were no significant differences in values of the eight waves when compared between the four investigators. However, the *F*-ratio and *p-*value of the S1 and alpha angle were not calculable by MedCalc for Wave04 and Wave07.

## Discussion

In this study, we proposed an algorithm for the digitization of time-based capnograph. We showed the step-by-step implementation in the previous sections yielding successful capnometric parameter extractions.

From the eight waves that were tested by four investigators, most of the parameters did not show a significant difference in values when the algorithm was applied. That meant that results were replicable and reproducible. We were not able to calculate both the S1 and alpha angle for two waves as mentioned in the results. As the alpha angle depended on the S1, this was the direct cause and implication of both parameters. We suspected a small sample size of waves and investigators being the cause of such occurrence. Furthermore, from the aforementioned waves, the possibility of missing pixels from binarization at the initiation of the breath resulted in both the S1 and alpha angle angles not being calculable (data not shown). The SNR should be optimized before thresholding to avoid missing pixels. In contrast, we did not encounter any *F*-ratios with significant differences between values that were obtained from the same waves.

## Limitations and Recommendation

The current limitation of the algorithm and its usage is that it needs further refinement. Further improvement could be made by shape recognition in future versions of the algorithm. Other than that, images that are captured with low megapixel cameras result in lower SNR resulting in a “salt and pepper” appearance which needs further refinement and extra signal processing steps. In this current algorithm, this issue has not been addressed yet as direct images from MATLAB were used. In this case, binarization was simple and we did not encounter special needs to utilize adaptive thresholding that is commonly used to address camera phone derived images.

Other than that, the images used for the capnographic signal digitization have both the x- and y-axis which show scales and values that could be used for the cropping part during the digitization process. However, real-time monitors in operation theater and intensive care units, usually display the absolute values for the y-axis but rarely display the time scale in seconds for the x-axis. This would result in the need to calculate the frequency and sampling rate before the conversion of the data into seconds. Furthermore, it is also logical to record the data in video form to calculate the exact time changes for the start and end of a breath signal before further processing.

We hope that this algorithm, which is the first step in capnographic digitization, would pave way for more capnometric indices, their clinical usages and, significance. It would be feasible in the setting where older capnographs in which direct signal processing is limited or impossible. Other than that, such digitization would also appeal to the current state of telemedicine as images are more common, convenient, fast, low-cost and, smaller in size when compared with the original capnographic signal.

Recommendations for future works would include the usage of “computer vision” in which real-time caponographic signals could be captured, digitized, and analyzed in real-time in a breath-to-breath manner prior to results display. This would urge more clinicians to be familiar with the established parameters as well as new parameters that can be further put to diagnostic and therapeutic use to aid clinical decision making.

## Conclusion

There is growing interest in the usage of capnograph away from its conventional utilization in the operation theater, intensive care unit, and emergency room. As more capnographic parameters are being derived and rediscovered by clinicians and researchers alike for both lung and non-lung-related diseases, there is a dire need for data analysis and interpretation. Although the proposed algorithm still needs minor refinements and further large-scale testing, we propose that the digitization of the capnograph *via* image processing technique could serve as an intellectual option as it is fast, convenient, easy to use, and efficient.

## Data Availability Statement

The raw data supporting the conclusions of this article will be made available by the authors, without undue reservation.

## Author Contributions

BV: original draft and writing—review and editing the manuscript. ES: data collection and reviewing the manuscript. MM: review and editing manuscript. All authors contributed to the article and approved the submitted version.

## Funding

This study was conducted as a part of the Research University Grant Scheme, supported by Universiti Teknologi Malaysia under Grant no: Q.J130000.2551.21H53.

## Conflict of Interest

The authors declare that the research was conducted in the absence of any commercial or financial relationships that could be construed as a potential conflict of interest.

## Publisher's Note

All claims expressed in this article are solely those of the authors and do not necessarily represent those of their affiliated organizations, or those of the publisher, the editors and the reviewers. Any product that may be evaluated in this article, or claim that may be made by its manufacturer, is not guaranteed or endorsed by the publisher.
